# The Mediating Role of Digital Competence in the Associations Between the Factors Affecting Healthcare Utilization and Access to Care

**DOI:** 10.3389/ijph.2023.1606184

**Published:** 2024-01-05

**Authors:** Tarja Heponiemi, Anu-Marja Kaihlanen, Lotta Virtanen, Emma Kainiemi, Petra Saukkonen, Päivikki Koponen, Seppo Koskinen, Marko Elovainio

**Affiliations:** ^1^ Finnish Institute for Health and Welfare, Helsinki, Finland; ^2^ Department of Psychology and Logopedics, University of Helsinki, Helsinki, Finland

**Keywords:** health services accessibility, digital competence, causal mediation analysis, longitudinal study, disadvantaged groups

## Abstract

**Objectives:** To examine with a population-based longitudinal survey design whether poor health, longstanding activity limitation, impaired cognitive functioning, mental distress, or loneliness predict poor access to healthcare and whether digital competence mediates these associations.

**Methods:** The data were from the longitudinal FinHealth -survey gathered in Finland in 2017 and 2020 including 3,771 respondents (57.1% women). Linear regression analyses were used to examine the associations of factors affecting healthcare utilization with access to care adjusted for age, sex, and education. Counterfactual causal mediation framework was used to examine the mediating role of digital competence in the relationships among these factors and access to healthcare.

**Results:** Factors affecting healthcare utilization were associated with poor access to care and these associations were partly mediated by low digital competence. Low digital competence mediated 12%, 9% and 8%, of the associations of impaired cognitive functioning, longstanding activity limitation, and loneliness with poor access to care, respectively.

**Conclusion:** According to our results, one way to improve the access to healthcare among vulnerable groups could be to improve their digital competence.

## Introduction

Poor access to healthcare and long waiting times are severe challenges in many countries [1]. One way that countries have tried to solve these problems is digitalization of healthcare services and these processes have been accelerated by COVID-19 pandemic restrictions [2–5]. Digital health services have been essential in maintaining access to care during the pandemic [6]. However, the need to use the internet to reach health services poses a substantial challenge because it can deepen inequalities in access to care between those who have the competence to use the internet and those who have not [7]. Thus, the increased delivery of digital health services could hinder some individuals from receiving healthcare services they need. In previous studies, low digital competence has, indeed, been associated with lower use of digital services [8, 9], thus it may predispose to poor access to care when health services are increasingly delivered online.

The Conceptual Framework of Access to Healthcare by Levesque et al. [10] points out the importance of individuals’ abilities to perceive, seek, reach, pay for, and engage in healthcare. Digital competence may nowadays be crucial for perceiving, seeking and reaching healthcare when services are increasingly delivered online. This is supported by the Framework for Developing and Understanding Digital Competence in Europe (DigComp) [11] which defines identifying digital resources and needs, making decisions about the appropriate digital resources and solving theoretical problems through digital resources as important aspects of digital competence. Moreover, digital competence can be seen to refer to the confident, critical and responsible use of, and engagement with, digital technologies for learning, at work, and for participation in society [12].

In the European Union, 73 percent of men and 69 percent of women have reported that they have sufficient competence in using digital technologies [13]. There is a growing body of evidence showing that there are vulnerable groups whose digital competence may not be sufficient for using digital healthcare or other services. The digital competence seems to be lower among older adults compared to younger adults [8, 9]. In addition, those who are experiencing socioeconomic or health-related disadvantages are more likely to have lower digital competence compared to others [14, 15]. Declines in cognitive functioning have been associated with lower ability to use computers [16] and deficits in digital competence have also been found among those with severe mental illness [17].

Previous studies have shown that they are the same disadvantaged groups such as those with low socioeconomic status, health problems, mental illness, loneliness, and physiological or cognitive dysfunction as well as older adults, who are also more susceptible to poor access to healthcare compared to their more privileged counterparts [18–22]. For example, in Italy during the COVID-19 pandemic, it was found that care delays, reduction in emergency department access, and hospitalisations were more common among older adults with higher number of chronic diseases [22]. In addition, care delays and reduction in emergency department access was more common among older adults with anxiety and depressive symptoms [22]. One explanation for lower access to care among disadvantaged groups might be that they are not able to utilize the full potential of digital health services, given that telehealth, for example, has been found to increase access to care [23].

The present study aimed to examine the associations between various factors affecting healthcare utilization and poor access to primary healthcare among a large population-based sample. More specifically, we examined whether longstanding activity limitation, impaired cognitive functioning, poor health, mental distress, or loneliness measured in 2017 predicted poor access to care during the preceding 12 months, measured in 2020, adjusted for age, sex, and education. Moreover, since the previous findings indicate that digital competence is associated with digital health service use [8, 9], we examined the mediating role of low digital competence measured in 2020 in these associations.

## Methods

### Sample

FinHealth 2017 study is a comprehensive nationally representative survey covering several aspects of health and wellbeing measured by questionnaires and clinical measurements [24]. The longitudinal survey data was gathered in two waves in 2017 and 2020. The original study sample, representing the Finnish adult population, was drawn from the Finnish Population Register. The baseline data was collected between January and May 2017 with a one- and two-stage stratified sampling design, a random sample comprising individuals aged 18 years or older and living in mainland Finland (total sample *n* = 10,305) including also people living in institutions. A more detailed description can be found elsewhere [24]. Altogether 7,050 persons participated in 2017 (participation rate 63.8%) [24].

During the second wave of the COVID-19 pandemic between October 2020 and January 2021, an updated sample from the previous survey, (*n* = 9,580) excluding those who had passed away, moved abroad or refused any further contact, was invited for a follow-up survey [25]. It was possible to fill in the questionnaire on paper, online, or participate in a shorter telephone interview. Altogether 5,400 persons responded to the follow-up survey (response rate 56.4%).

In the present study, we included respondents who had responded to the questionnaire both in 2017 and 2020 (*n* = 4,881). We excluded those participants who had not used primary health services in the past 12 months or had missing information in any of our study variables, for example, because having participated only in a short telephone interview which did not cover all our study variables (1,100 were excluded). Thus, the present study included 3,771 respondents (57.1% women) aged between 22 and 98 years (Mean = 58.4, SE = 15.8) who had answered all survey questions. An Inverse Probability Weighting (IPW) correction based on register data on age, sex, marital status, education level, region of residence, language, and possible hospitalizations was used. Previous studies have shown the suitability of this method for correcting possible non-response bias among the Finnish population [26].

### Ethical Issues

Participation in the study was completely voluntary and the participants were provided with an opportunity to withdraw from the study at any time. The FinHealth 2017 Study has received approval from the Coordinating Ethics Committee at the Hospital District of Helsinki and Uusimaa (Reference 37/13/03/00/2016) and a follow-up study from the Ethics Committee II of the Helsinki and Uusimaa Hospital District (HUS/2391/2020).

### Measurements

#### Dependent Variables Measured at Follow-Up in 2020

Poor access to primary healthcare was measured by three items asking respondents to evaluate their experiences in their primary care facility in the past 12 months (not including dental care). The items were: “I was able to get contact to the unit without difficulty”; “I had access to care without undue delay”; and “I had access to examinations (laboratory tests, X-ray imaging, ultrasound scans, etc.) without undue delay.” A mean score was calculated from the response options 1 = always, 2 = usually, 3 = sometimes, and 4 = never, which was used as a continuous dependent variable in the analyses (reliability Cronbach’s alpha *α* = 0.85).

Low digital competence was assessed with a question asking the respondents’ assessment of their digital competence to use online services (on computer or mobile devices). The response options were a) I do not use them, b) novice/beginner, c) I can use the basic services independently, d) I can use many online services effortlessly and e) expert (I can teach others). This question has been used previously and associated for example, with internet-based service usage [27, 28]. The responses were coded as 0 = good or average competence (answer options c–e) and 1 = low competence (answer options a and b).

#### Factors Affecting Healthcare Utilization at Baseline in 2017

Longstanding activity limitation was measured with the two items Global Activity Limitation Indicator (GALI) which is a comprehensive survey instrument measuring physical restrictions [29, 30]. The first part of the question was “Are you limited because of a health problem in activities people usually do? Would you say you are…” with three response alternatives: a) severely limited, b) limited but not severely, or, c) not limited at all. If the respondent indicated being limited, the question continued as “Have you been limited for at least the past 6 months?” (yes/no). Those with a limitation (first question’s options a and b) that had lasted at least 6 months (second question, option “yes” were classified as having longstanding activity limitation. The measure was coded as 0 = no longstanding activity limitation and 1 = longstanding activity limitation.

Impaired cognitive functioning was measured with three items assessing how respondents estimated their present memory, learning capabilities, and ability to concentrate [31] (*α* = 0.80). The items included five response options: a) very well, b) well, c) adequately, d) poorly, and e) very poorly. Those who had chosen answer options from c to e to all three questions were rated as having impaired cognitive functioning. The measure was coded as 0 = normal cognitive functioning and 1 = impaired cognitive functioning.

Poor health was measured by a widely used question from previous national health surveys in Finland: “How is your present state of health?”, with the response options a) good, b) rather good, c) moderately good, d) rather poor, and e) poor. This wording differs from the widely used European Health Interview survey question [32] but was chosen for the FinHealth 2017 Study to follow national time trends [33, 34]. Self-rated health has been shown to demonstrate a strong and logical association with numerous objective biomarkers showing that it is a robust and comprehensive indicator of health-related processes [33]. The measure was coded as 0 = good health (response options a and b) and 1 = poor health (response options c–e).

Mental distress was assessed with five items from the Mental Health Inventory (MHI-5) derived from the SF-36 scale [35]. MHI-5 has been found easy to fill, valid, and reliable for use in different settings and groups [36, 37]. MHI-5 includes five items covering the past 4 weeks related to feelings of nervousness, downness, calmness, downheartedness, and happiness. All items were rated on a 6-point system ranging from “All of the time” to “None of the time.” The items related to calmness and happiness were reversed coded. The sum scores (*α* = 0.89) were transformed to a scale ranging from zero to 100 [38]. As suggested [38], a total score of 52 or below was considered the cut-off point for current mental distress. The measure was coded as 0 = no mental distress (>52 points) and 1 = mental distress (≤52 points).

Loneliness was assessed with a question “Do you ever feel lonely?” The response options were a) never, b) very rarely, c) sometimes, d) fairly often, and e) all the time. This measure has been found to be appropriate for measuring loneliness [39] and results from previous studies suggest that it may serve as a direct indicator of the feeling of loneliness [40, 41]. The measure was coded as 0 = no (options a and b) and 1 = yes (options c–e), which is similar to what has been previously used [41].

#### Adjustment Variables

Age (in 2020) and *sex* were obtained from the National Population Register. Education was coded as 0 = upper secondary or higher education and 1 = lower secondary or less education.

### Statistical Analysis

Linear regression analyses were used to examine the associations of the explanatory variables (2017) with poor access to care (2020). Logistic regression analyses were used to examine the associations of the explanatory variables with low digital competence. Both analyses were conducted in two steps. First, we examined the univariable associations between each independent variable and dependent variables (Models A). Second, we examined the multivariable associations for dependent variables by simultaneously including all the studied independent variables in the analyses, adjusted for age, sex, and education (Models B). Thus, the multivariable model for poor access to care included longstanding activity limitation, impaired cognitive functioning, poor health, mental distress, loneliness, low digital competence, and adjustment variables. The multivariable model for low digital competence included longstanding activity limitation, impaired cognitive functioning, poor health, mental distress, loneliness, and adjustment variables.

To examine the mediating role of digital competence in the relationships among independent variables and poor access to healthcare, we used a counterfactual causal mediation framework using the simulation-based Monte-Carlo approach with the sequential ignorability assumption [42]. The counterfactual approach includes examining whether access to care changes under conditions in which factors affecting healthcare utilization and digital competence are manipulated to represent relevant counterfactual scenarios [42–44]. The effects were separated into average causal mediation (indirect) effects, average direct effects, and total effects. To obtain the confidence intervals, bootstrapping was performed for each of the models with 1,000 replications. Methods suitable for weighted data were used. Causal mediation analyses were conducted with the low digital competence as a potential mediator adjusting for age, sex, education, and independent variable. Each independent variable (longstanding activity limitation, impaired cognitive functioning, poor health, mental distress, and loneliness) was examined in separate analyses. We used the causal inference methods for mediation analysis (“causal mediation”) which is an extension of the traditional approach, developed to better address for effect decomposition in the presence of exposure-mediator interaction and to clearly explicate the four main assumptions for estimating direct and indirect effects, including no unmeasured confounding assumptions. All the analyses were conducted using R statistical software version 4.2.1 and the causal mediation with R package Mediation [42, 45].

## Results

Characteristics of the study sample can be seen in Table 1. The mean age of the sample was 58 years and there were more females (57.1%) than males. The majority of the respondents had high or upper secondary education, whereas less than one in five had no more than lower secondary education. Approximately two in five had low digital competence. Similarly, two in five of the respondents experienced longstanding activity limitations. Around one in three perceived their health as poor, and similarly reported feelings of loneliness. The minority of the respondents reported impaired cognitive functioning and mental distress.

**TABLE 1:** Characteristics of the study sample, unweighted (N = 3,771). (FinHealth -survey, Finland, 2017 and 2020).

Variable	Mean (SD)/n(%)
Age^a^, Mean (SD)	58.4 (15.8)
Poor access to care^b^, Mean (SD)	1.58 (0.611)
Sex, n (%)	
Male	1,618 (42.9%)
Female	2,153 (57.1%)
Education, n (%)	
Secondary and high education	3,148 (83.5%)
Low education	623 (16.5%)
Digital competence, n (%)	
High or moderate	2,164 (57.4%)
Poor	1,607 (42.6%)
Longstanding activity limitation, n (%)	
No limitations	2,350 (62.3%)
Longstanding activity limitation	1,421 (37.7%)
Cognitive functioning, n (%)	
Normal cognitive functioning	3,267 (86.6%)
Impaired cognitive functioning	504 (13.4%)
Self-rated health, n (%)	
Normal	2,470 (65.5%)
Poor	1,301 (34.5%)
Mental health, n (%)	
Normal	3,522 (93.4%)
Mental distress	249 (6.60%)
Loneliness, n (%)	
No	2,696 (71.5%)
Yes	1,075 (28.5%)

^a^ Ranged between 21.8 and 98.1.

^b^ Ranged between 1 and 4.

Univariable linear regression analyses (Table 2, Model A) showed that women and those who experienced that they have longstanding activity limitation, impaired cognitive functioning, poor health, mental distress, loneliness, and low digital competence were more likely to perceive that they have poor access to primary healthcare. In the multivariable analysis (Table 2, Model B), sex, impaired cognitive functioning, poor health, and low digital competence remained significant predictors of poor access to care.

**TABLE 2:** Results of linear regression analyses for poor access to care (N = 3,771). (FinHealth -survey, Finland, 2017 and 2020).

Variable	Model A^a^			Model B^b^		
	b	95% CI	*p*-value	b	95% CI	*p*-value
Age	0.00	−0.00 to 0.00	0.147	−0.00	−0.00 to 0.00	0.026
Sex	0.11	0.06 to 0.16	<0.001	0.10	0.05 to 0.16	<0.001
Low education	0.07	−0.00 to 0.13	0.050	−0.01	−0.08 to 0.06	0.766
Longstanding activity limitation	0.16	0.11 to 0.21	<0.001	0.04	−0.01 to 0.11	0.123
Impaired cognitive functioning	0.22	0.14 to 0.29	<0.001	0.10	0.03 to 0.17	0.008
Poor health	0.23	0.18 to 0.28	<0.001	0.17	0.10 to 0.23	<0.001
Mental distress	0.25	0.12 to 0.37	<0.001	0.10	−0.03 to 0.23	0.118
Loneliness	0.13	0.07 to 0.20	<0.001	0.06	−0.00 to 0.12	0.061
Low digital competence	0.14	0.09 to 0.19	<0.001	0.12	0.06 to 0.17	<0.001

^a^ Univariable analyses.

^b^ Multivariable analyses including all studied variables.

b, unstandardized regression coefficient; CI, confidence interval.

The univariable logistic regression analyses (Table 3, Model A) showed that older respondents and those with low education, longstanding activity limitation, impaired cognitive functioning, or poor health had greater odds of low digital competence compared to their counterparts. In the multivariable logistic regression (Table 3, Model B), all the other variables except longstanding activity limitation remained significant predictors.

**TABLE 3:** Results of logistic regression analyses for low digital competence (N = 3,771). (FinHealth -survey, Finland, 2017 and 2020).

Variable	Model A^a^			Model B^b^		
	OR	95% CI	*p*-value	OR	95% CI	*p*-value
Age	1.10	1.09–1.11	<0.001	1.08	1.08–1.09	<0.001
Sex	1.10	0.94–1.29	0.233	1.08	0.89–1.29	0.440
Low education	6.38	4.85–8.39	<0.001	2.71	2.09–3.54	<0.001
Longstanding activity limitation	2.84	2.41–3.34	<0.001	1.18	0.97–1.43	0.106
Impaired cognitive functioning	6.03	4.60–7.89	<0.001	2.42	1.85–3.18	<0.001
Poor health	3.66	3.08–4.35	<0.001	1.69	1.37–2.08	<0.001
Mental distress	1.16	0.86–1.57	0.318	0.89	0.61–1.29	0.539
Loneliness	1.19	1.00–1.42	0.055	1.23	0.98–1.55	0.068

^a^ Univariable analyses.

^b^ Multivariable analyses including all studied variables.

OR, odds ratio; CI, confidence interval.

The associations of longstanding activity limitation, impaired cognitive functioning, poor health, mental distress, and loneliness with poor access to care were all partially mediated through low digital competence (Table 4). Approximately 12% of the association between impaired cognitive functioning and poor access to care was estimated to be mediated through low digital competence. In the other associations, the mediating role of low digital competence was smaller; 9% of the association of activity limitation, 8% of the association of loneliness, 6% of the association of poor health and 4% of the association of mental distress with poor access to care.

**TABLE 4:** The results of the mediation analyses with low digital competence as mediator for poor access to care (N = 3,771). (FinHealth -survey, Finland, 2017 and 2020).

Parameter	*b*	95% CI	*p-*value
Longstanding activity limitation as independent			
Average natural mediated effect	0.01	0.01–0.02	<0.001
Average natural direct effect	0.14	0.08–0.19	<0.001
Total effect	0.15	0.10–0.20	<0.001
Proportion mediated	0.09	0.03–0.14	<0.001
Impaired cognitive functioning as independent			
Average natural mediated effect	0.03	0.01–0.04	<0.001
Average natural direct effect	0.19	0.11–0.26	<0.001
Total effect	0.21	0.14–0.29	<0.001
Proportion mediated	0.12	0.07–0.22	<0.001
Poor self-rated health as independent			
Average natural mediated effect	0.01	0.01–0.02	<0.001
Average natural direct effect	0.22	0.17–0.26	<0.001
Total effect	0.24	0.19–0.28	<0.001
Proportion mediated	0.06	0.03–0.10	<0.001
Mental distress as independent			
Average natural mediated effect	0.01	0.00–0.02	0.02
Average natural direct effect	0.23	0.15–0.30	<0.001
Total effect	0.24	0.16–0.32	<0.001
Proportion mediated	0.04	0.01–0.09	0.02
Loneliness as independent			
Average natural mediated effect	0.01	0.00–0.02	<0.001
Average natural direct effect	0.11	0.07–0.16	<0.001
Total effect	0.12	0.08–0.17	<0.001
Proportion mediated	0.08	0.04–0.15	<0.001

Models adjusted for age, sex and education. Abbreviations: *b*, Unstandardized regression coefficient; CI, Confidence interval.

## Discussion

The present study found that longstanding activity limitation, impaired cognitive functioning, poor health, and mental distress predicted poor access to primary healthcare three to four years later and these associations were partially accounted for by low digital competence. Digital competence mediated most the association between impaired cognitive functioning and poor access to care.

Our results suggest that disadvantaged groups such as those with physical or cognitive limitations, poor physical or mental health, and loneliness are at risk of poor access to healthcare, congruent with previous studies [18–22]. Previous studies also show that those with impairments in health or memory or low levels of social relations tend to use less computer and digital services and perceive that they benefit less from them [7, 28, 46, 47]. Our findings and previous findings are worrying given that the people in these disadvantaged groups need healthcare the most. There is a substantial risk that these vulnerable people do not receive the health services they need and the increased delivery of digital services aggravates this inequal situation.

Previous studies have shown that many people with disabilities are dependent on the support and encouragement provided by professionals in the use of information technology and the internet [48]. With appropriate support, most people for example, with mild or moderate cognitive disabilities can learn the basic skills required to use information technology [49]. It has also been shown that people with more severe intellectual disabilities can use information technology to a limited extent when receiving high-quality support [50]. Support should be provided in multiple channels at a low threshold. In addition, the individual’s ability to use technology and digital services should not be underestimated, which can lead to the customer not being offered digital services as an opportunity [48].

Our results show that in increasingly digitalized services digital competence plays an important role in the access to healthcare, given that digital competence partly accounted for the association between factors affecting healthcare utilization and poor access to care. Digital competence had the strongest role in the association between impaired cognitive functioning and poor access to care. This finding complements previous findings showing that impaired cognitive functioning is associated with lower ability to use computers [16] and lower internet use [46]. Our results suggest that those disadvantaged people who have problems with health, functioning or social relations and additionally have low digital competence are particularly in danger of not receiving the health services they need.

According to our results, the increase in providing digital services and the need for digital competence may reinforce inequalities in access to healthcare. A previous study suggests that telemedicine is not likely to provide equal access to clinical care for all sections of the population such as economically, racially, ethnically, and socioeconomically vulnerable groups [51]. Disparities based on mental wellbeing have been found in mobile health medication adherence promotion interventions [52]. Similarly, inequalities in access to digital health services has been found among those with low digital health literacy skills, low socioeconomic status, and low education [53].

### Strengths and Limitations

The strengths of our study were a longitudinal design and a national population-based sample with a fairly good participation rate. However, our study includes some limitations which should be considered when interpreting the findings. Three measurement points would have been preferable for the causal mediation -test. Our data is based on self-reported data, which can lead to problems associated with common method variance and inflation of the strengths of associations. To correct for possible response bias, we used IPW correction [26] based on age, sex, marital status, education level, and region of residence. Previous studies have shown an IPW method suitable for adjusting possible non-response bias among the Finnish population [26]. We controlled for age, sex, and education; however, a possibility of residual confounding still exists. In addition, our measure of digital competence included only one general question, thus it did not cover all dimensions that digital competence has been suggested to include [12]. Thus, future studies should examine more precisely which dimensions of digital competence are of the greatest importance in this context. Moreover, generalising our findings to other countries with different kinds of healthcare systems and digitalization levels should be done with caution, because Finland is a forerunner in the digitalisation of services.

### Conclusion

The present study showed that physically, mentally, or socially disadvantaged people are in danger of poor access to care and poor digital competence partly accounts for this. Thus, our results support Levesque et al.’s [10] and DigiComp [11] frameworks and highlight the importance of the ability to seek, reach, and engage in digital healthcare. Our results suggest that one way to increase access to care among vulnerable groups is to increase their digital competence. Moreover, digital service providers should focus on the usability and clarity of their services and, thus, make them easy to use also for those with less digital competence. It is important that authorities can identify those with poor digital competence and implement interventions aiming to improve their digital competence. It is important to ensure that people get access to healthcare services irrespective of their level of digital competence. For example, ICT training, which includes practicing in pairs, the possibility to practice with the device, the possibility to influence what to learn, good communication, and the availability of helping material has been found useful in improving digital competence [54]. In addition, it has been found that when teaching the use of the computer and the internet, it is important to tie education to the individual’s current competence and goals and to ensure the supportive teaching environment [55]. However, many people are not able to use digital services and it is of utmost importance to guarantee services to them as well.
